# A machine learning framework for the quantification of experimental uveitis in murine OCT

**DOI:** 10.1364/BOE.489271

**Published:** 2023-06-16

**Authors:** Youness Mellak, Alin Achim, Amy Ward, Lindsay Nicholson, Xavier Descombes

**Affiliations:** 1 Université Côte d’Azur, INRIA, CNRS, I3S, Sophia Antipolis, France; 2 University of Bristol, Bristol, United Kingdom

## Abstract

This paper presents methods for the detection and assessment of non-infectious uveitis, a leading cause of vision loss in working age adults. In the first part, we propose a classification model that can accurately predict the presence of uveitis and differentiate between different stages of the disease using optical coherence tomography (OCT) images. We utilize the Grad-CAM visualization technique to elucidate the decision-making process of the classifier and gain deeper insights into the results obtained. In the second part, we apply and compare three methods for the detection of detached particles in the retina that are indicative of uveitis. The first is a fully supervised detection method, the second is a marked point process (MPP) technique, and the third is a weakly supervised segmentation that produces per-pixel masks as output. The segmentation model is used as a backbone for a fully automated pipeline that can segment small particles of uveitis in two-dimensional (2-D) slices of the retina, reconstruct the volume, and produce centroids as points distribution in space. The number of particles in retinas is used to grade the disease, and point process analysis on centroids in three-dimensional (3-D) shows clustering patterns in the distribution of the particles on the retina.

## 1. Introduction

The realm of using deep learning (DL) in image recognition, specifically convolutional neural network (CNN), exhibits significant potential in multiple medical tasks. Ophthalmology is not an exception, as it relies heavily on high-precision imaging techniques for both diagnosis and monitoring of disease progression. In fact, artificial intelligence (AI) initiatives have already been implemented for various ophthalmic conditions such as glaucoma [1–4], Age-Related Macular Degeneration [5–12], and Retinopathy of Prematurity [13–15] with remarkable accuracy.

Non-infectious uveitis is an inflammatory disease that affects the vascular uveal tract of the eye and can lead to serious clinical complications in humans [16]. The condition can involve multiple ocular structures, including the retina, which can result in severe visual impairment. Uveitis is a major cause of vision loss in individuals aged 20 to 60 years, ranking fifth in prevalence. In the United States alone, it is responsible for more than 10% of cases of severe visual impairment [17]. To better understand the pathogenesis of uveitis and evaluate new therapeutic and diagnostic techniques, experimental autoimmune uveitis (EAU) is widely used as animal model for ocular autoimmunity. EAU shares essential pathological features with human uveitis and can be induced by active immunization with retinal proteins or their peptides of susceptible mouse strains or by transferring ocular tissue-specific CD4+ T lymphocytes into naive recipients. Therefore, EAU is an effective model for studying the mechanisms underlying human uveitis and for evaluating the effectiveness of new therapies and diagnostic methodologies [18,19].

Accurate detection and monitoring of disease is a crucial component of the medical treatment of human uveitis. It is widely accepted that OCT is the most promising approach for quantification of uveitis and useful attempts to define the characteristics of active disease have been reported [20]. Nevertheless, most current assessments remain subjective and automated algorithms are found in some cases to be less robust in the presence of inflammation [21]. The development of automated tools to objectively and precisely characterize the pathological changes induced by uveitis is the subject of this work. This remains a challenging and important goal in posterior uveitis.

The first part of our work focuses on applying deep learning to classify ill retinas from 2D images in an early stage of the disease and before the ophthalmologists themselves can detect the symptoms of the disease in OCT. In addition, we apply a technique that produces a visual explanation of what pushes the convolutional neural network to make a particular decision.

In the second part, we apply different methods for object detection and segmentation and notably, we are not limiting ourselves to only obtaining bounding boxes. Instead, we define the object detection task as getting a single set of 2-D coordinates corresponding to the location of each object. The location of an object can be any key-point, such as its centre. Unlike other key-points detection problems, we do not know in advance the number of points in a slice taken from a retinal image. To also make the description of the problem as generic as possible, we do not assume any constraints between points, unlike cases such as pose estimation, as described in [22]. This definition of object location is more appropriate for an application such as ours, where the objects are very small and/or overlap. To evaluate the results of the proposed method, we compare it to one of our MPP methods then we trained Faster R-CNN [23] on our database with bounding boxes as an annotation.

The final part of this article shows how we can take advantage of the segmentation of retina and particles to extract some important information from retinas on individual days of the analysis or to perform a point process analysis to study the distribution.

## 2. Material and methods

### 2.1 Deep learning approach for classification and explainability

In this section, our aim is to perform a classification task that involves comparing pairs of images captured on different days, as well as performing a collective classification across all available days. In addition, we use an explainable AI methodology to gain a comprehensive understanding of the classification outcomes and illuminate the underlying factors that contribute to the results obtained.

#### 2.1.1 EfficientNet-B7

The theoretical foundation of deep learning posits that the use of deep architectures leads to the extraction of increasingly detailed features, thereby improving classification performance. However, empirical evidence has demonstrated that after a certain number of layers, the performance of such architectures begins to deteriorate. To address this issue, He et al. [24] proposed the *residual block*, which establishes a direct connection, or a "skip" connection, between the beginning and the end of a convolution block. This allows the architecture to retain features from earlier layers, mitigating overfitting. However, this technique suffers from two drawbacks, namely, an increase in the number of layers and the high memory usage associated with storing and summing large volumes of characteristics. In response, Tan et al. [25] introduced the *inverted residual block*, which utilizes features with fewer channels, and links them together in a "narrow -> wide -> narrow" analogy.

Beyond simply optimizing the architecture to achieve greater depth with reduced memory usage, Tan et al. [25] proposed a method for automatically controlling the three dimensions of an architecture (i.e., depth, width, and resolution). This approach is employed in the EfficientNet-B0 architecture introduced in the same work. The proposed method utilizes a coefficient, denoted as ψ , which regulates the three dimensions to achieve a balance between model accuracy and computational efficiency. Specifically, the number of layers, d , is given by $d=\alpha^{\psi }$ , the number of filters, w , is given by $w=\beta^{\psi }$ , and the resolution of the input image, r , is given by $r=\gamma^{\psi }$ . These dimensions are subject to two conditions: i) $\alpha \times \beta^{2}\times \gamma^{2}\approx 2$ , which ensures that the increase in architecture is proportional to an increase in floating point operations per second (FLOPS) with a scaling factor of $2^{\psi }$ , and ii) α≥1,β≥1,γ≥1 , which guarantees an increase in the dimension in question. In the present work, we consider the EfficientNet-B7 architecture.

#### 2.1.2 Grad-CAM

**Fig. 1.**
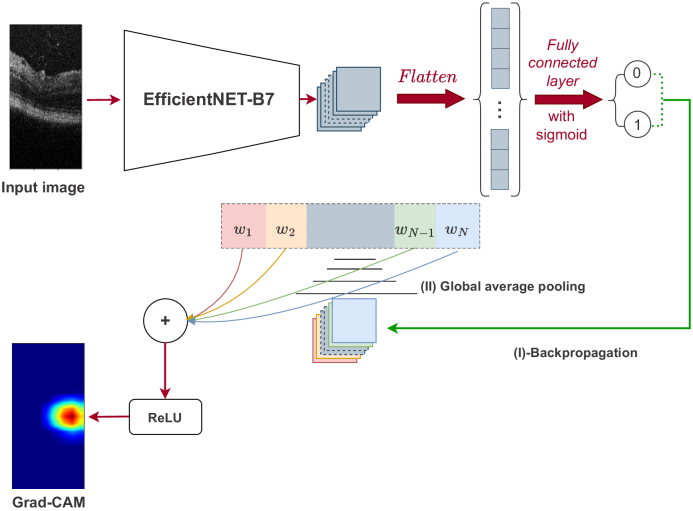
The pipeline of gradient-weighted class activation mapping (Grad-CAM). The input image is fed to a trained neural network (EfficientNET-B7) in order to obtain the classification result. Back propagation is performed with ill retina = 1 and healthy retina = 0. GAP of the gradient is calculated for each channel and used as weights for the network. The weights are then multiplied with the feature map, summed and passed to the ReLU to obtain the heatmap.

Deeper representations in CNNs can capture higher-level visual structures, which can help to identify more complex visual patterns. In addition, convolutional layers in CNNs preserve spatial information that is lost in fully connected layers, thus achieving the best compromise between high-level semantics and detailed spatial data information. The final convolutional layer in a CNN is particularly important because neurons in this layer look for semantic class-specific information in the image. Gradient-weighted class activation mapping (Grad-CAM), introduced by Selvaraju et al. [26], is a technique that uses gradient information flowing into the last convolutional layer of the CNN to assign importance values to each neuron for a specific class of interest. This technique is general and can be used to explain the activations of any layer of a deep neural network. In our work, we use Grad-CAM to obtain insights into how the network is processing images, Fig. 1 summarize the workflow for obtaining the heat-maps.

### 2.2 Particle detection

The standardized numerical grading of cells or flare observed during slit-lamp examination by ophthalmologists using the standardization of uveitis nomenclature (SUN) grading system is currently the widely recognized gold standard method for evaluating the severity of anterior uveitis [27]. However, developing automated approaches capable of achieving similar levels of accuracy and reliability can significantly enhance the diagnostic and decision-making processes associated with this condition. In the ensuing section, we utilize three distinct approaches, ranging from MPP to fully supervised techniques, to detect and/or segment uveitis particles. Furthermore, we extend the detection process to extract the volume of each particle in 3-D. This additional information can serve as a valuable tool in further exploring the underlying characteristics of the disease, as we will demonstrate.

#### 2.2.1 Supervised object detection

**Fig. 2.**
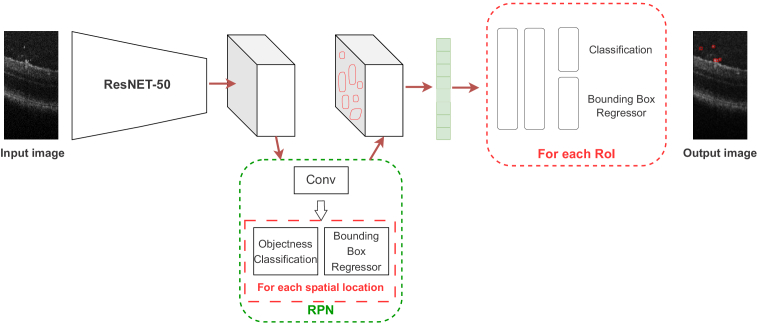
Faster R-CNN to predict bounding boxes around the particles.

Faster R-CNN, a CNN architecture proposed by Ren et al. [23], is a powerful tool for detecting objects of interest within images. The network is composed of two fundamental components: a CNN backbone, which serves to extract high-level features from the image, and a region proposal network, which generates high-quality proposals for object regions within the image, see Fig. 2. The latter component leverages another CNN to simultaneously perform object boundary regression and objectness classification for each proposal. The resulting proposals are subsequently utilized to accurately identify the location of objects of interest, which are assigned class labels. In our case, a pre-trained ResNet50 [24] architecture is employed as the backbone to extract features.

#### 2.2.2 Weakly supervised segmentation

**Fig. 3.**
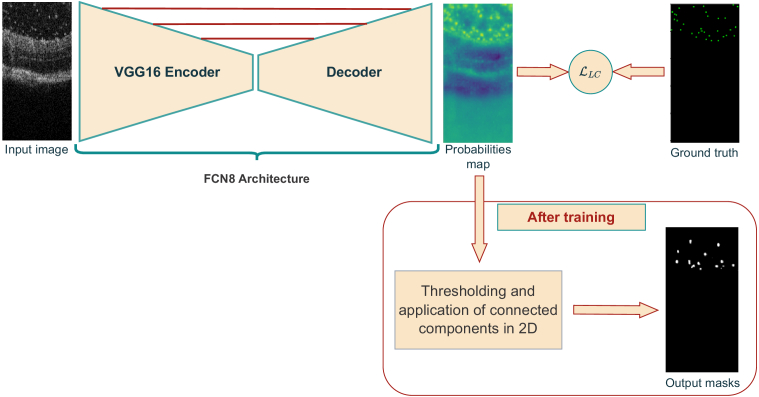
Weakly supervised segmentation with LC-FCN. 2-D OCT images are used as input. The FCN8 architecture is used to generate probability maps. These represent the probability of each pixel being part of a particle. The output of FCN8 is thresholded by 0.5 and then passed to a 2-D connected components algorithm to obtain the masks and corresponding number of particles.

In the deep learning literature, convolutional network techniques, specifically the hourglass architecture complemented by an augmented loss function, have been identified as an effective means of determining both the number and location of objects using only point annotations [28–30]. In our work we used the method proposed by Laradji et al. [31], where the authors use an hourglass architecture to map an image to a matrix of probabilities that represent the probability of an pixel to belongs to an objects or the background. The novelty of the work is the use of new loss that consists of four terms: image-level loss adjusts the model to determine that at least one pixel of the image belongs to each class present in the image, while Point-level loss encourages it to correctly identify pixels with point annotations. Split-level loss discourages the model from predicting blobs with two or more point annotations, while False Positive loss reduces the number of false positive predictions in the model’s output. Figure 3 shows the workflow of the method to predict particles.

#### 2.2.3 MPP for object detection:

To detect particles using conventional techniques we consider the marked point process framework to fit a set of vertical rectangles on the brightest spots on each 2D image of the retina stack [32]. The detection was performed with the software ObjMPP [33]. We estimate a collection of non overlapping rectangles. Each object exhibits a contrast with its neighborhood evaluated by the normalized difference between pixel means within the rectangle and the crown surrounding it respectively.

Consider the image $I=\{i_{s}\in \Lambda ,s\in L\}$ , where Λ⊂N is the grey level set and $L\subset R^{2}$ is the image plane. A vertical rectangle is defined by $\left\{r,w,l\}\in R,R=L\times [w_{min},w_{max}]\times [l_{min},l_{max}\right]$ , w , resp. l , representing the width, resp. the length, of the rectangle.

A configuration is a set of rectangles:

(1) $$\omega =\{r_{i},i\in \{1,n\},r_{i}\in R\}\in \Omega .$$

The detection result is the configuration minimizing the following energy function:

(2) $$E=\sum_{i\in \{i,n\}}D(r_{i})+\sum_{i,j\in \{i,n\}\times \{1,n\}}O(r_{i},r_{i})$$

where $D(r_{i})$ is the data term given by:

(3) $$D(r_{i})=Q(x)$$

with Q a quality function defined as follows :

(4) $$Q(x)=\left\{ \begin{array}{l} 1-\frac{x}{x_{0}}\;if\;x<x_{o}\\ \exp\left(\frac{-(x-x_{0})}{x_{0}}\right)\;otherwise \end{array}\right.$$

$x_{0}$ begin a threshold value and

(5) $$x=\frac{{\left(\mu (r_{i})-\mu (d(r_{i}))\right)}^{2}}{\sqrt{\sigma^{2}(r_{i})+\sigma^{2}(d(r_{i}))}}$$

where $\mu (r_{i})$ (resp. $\sigma^{2}(r_{i})$ ) is the mean value (resp. the variance) of pixels in the rectangle $r_{i}$ , and $\mu \left(d(r_{i}\right))$ (resp. $\sigma^{2}(d(r_{i}))$ ) is the mean value (resp. the variance) of pixels in the neighborhood of the rectangle $r_{i}$ .

$O(r_{i},r_{j})$ is the non overlapping term:

(6) $$O(r_{i},r_{j})=\left\{ \begin{array}{l} 0\;if\;r_{i}\cap r_{j}=\emptyset \\ \infty \;otherwise \end{array}\right.$$

The energy minimization is performed using the multiple births and cut algorithm [34]. The parameters have been tuned on three images taken from three different stacks. The same parameter values have then been used on the whole datasets.

### 2.3 Statistical analysis

This section involves utilizing 3-D masks of uveitis particles to perform a statistical analysis of their cluster patterns in the retina. Additionally, we use a CNN network to detect the retina surface, which can facilitate the investigation of particle distribution patterns in relation to the retinal surface.

#### 2.3.1 Extraction of the retinal surface

**Fig. 4.**
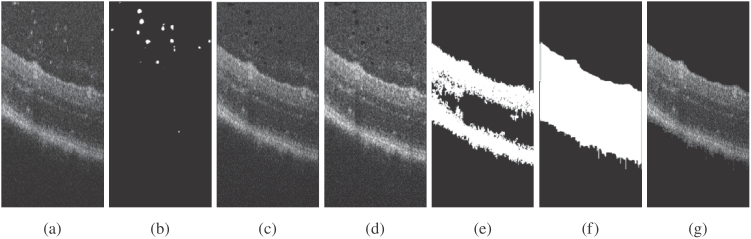
A multi-step image processing approach for extracting the retina surface from an OCT image. (a) Original image, (b) Extracted masks of particles, (c) Image without particles, (d) Normalization of grayscale on small columns (of 10 pixels) of the image, (e) Binarization with a threshold, Application of connected components algorithm in 2-D and removing small regions, then smoothing image with Gaussian filter, (f) Extracted retina mask, (g) Extracted retina surface.

The community has focused on developing automated software to segment distinct retinal layers in OCT images of the mouse eye, as evidenced in previous works [35–37]. However, in our current study, our objective is limited to obtaining a mask for all retina surfaces in 2D, rendering a more targeted approach suitable. Due to the unavailability of ground truth data, a MPP method was adopted. Since OCT images of the mouse retina are often afflicted by noise, poor resolution and particles or artefacts near the surface of the retina, the task at hand is challenging. The proposed method relies on classical image processing techniques and involves the heuristically-based pre-setting of certain parameters, such as the image threshold, which is used to achieve image binarization. The steps for retinal surface extraction from OCT images are depicted in Fig. 4.

**Fig. 5.**
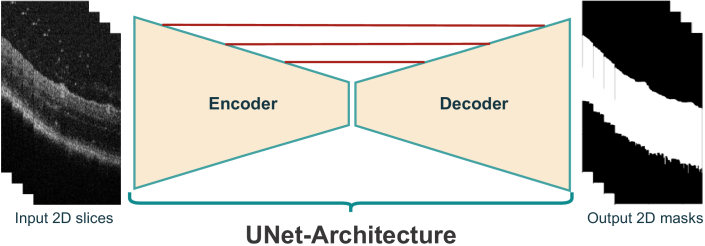
Deep Learning-Based Retina Surface Extraction using U-Net.

As classical image processing techniques are not robust to variability in contrast and gray scale, modification of thresholds and parameters of the previous technique is a must. For that we used the generated masks by the classical approach to train a UNet architecture [38] to map OCT images to masks of the retina (Fig. 5).

#### 2.3.2 Particle distribution

**Fig. 6.**
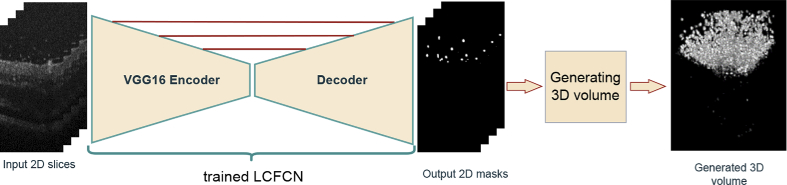
Pipeline to generate 3-D distribution of particles. Generating 3-D volume step include gathering 2D slices in a unique volume, followed by application of 3-D connected components algorithm and shape Filtering to enhance particle detection.

Extracting particle distribution in 3-D is the base for any study on particle distribution or movement in the retina. Figure 6 summarize our proposed approach. First, we pass all slices of the retina to our segmentation algorithm (LCFCN), accuracy is enhanced by letting just particles inside a bounding box given by Faster R-CNN. Then we construct a 3-D volume of particles, on which we apply connected components in 3-D to create a label on each whole particle. The final step consists in extracting a centroid of each element to obtain a points distribution.

The subsequent subsection provides an illustrative example of the functions that can be utilized to study the patterns of uveitis distribution. Specifically, we employ k-ripley in 3-D to investigate the clustering of uveitis particles across multiple days.

**Fig. 7.**
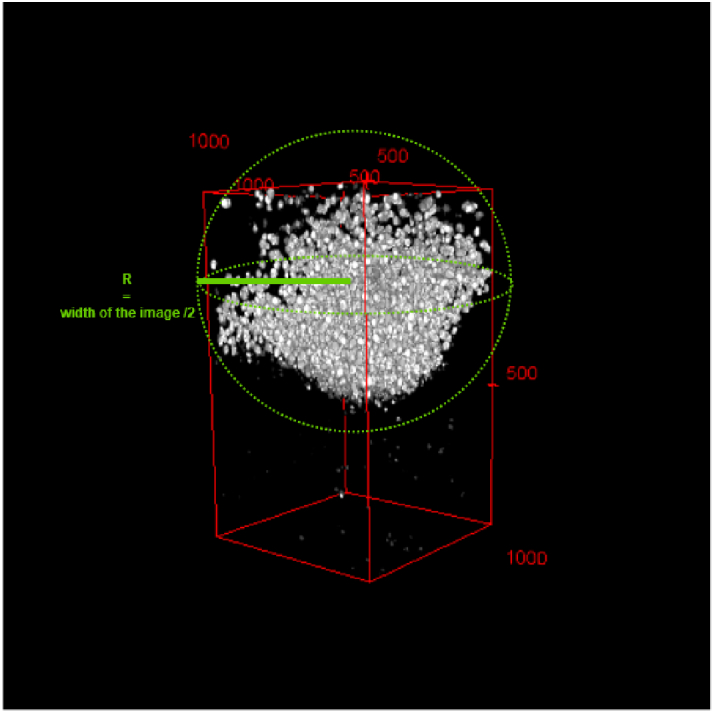
The image of segmented particles in 3-D is in white, the volume of the entire retina is in red, and the studied area where the K-Ripley function is calculated is the sphere in green.

#### 2.3.3 Clustering index (K-Ripley function):

Ripley’s K-function is a numerical tool for evaluating the structure of the underlying point pattern in a sample. Its non-parametric nature means that it is independent of prior knowledge about the distribution family of samples. Regardless of the domain to which it is applied, Ripley’s K-function can be expressed as:

(7) $$K(r)=\frac{W}{n(n-1)}\sum_{i}\sum_{j\neq j}I\{\left\|x_{i}-x_{j}\right\|\leq r\}c(x_{i},x_{j},r),$$

where n is the total number of points in the observation window, $I\{\left\|x_{i}-x_{j}\right\|\leq r\}$ is an indicator function which is worth 1 if points i and j are at a distance at most equal to r and 0 otherwise, and c( $x_{i}$ , $x_{j}$ ;r) corresponds to the correction of edge effects proposed in [39] and W to the study area represented in Fig. 7.

**Fig. 8.**
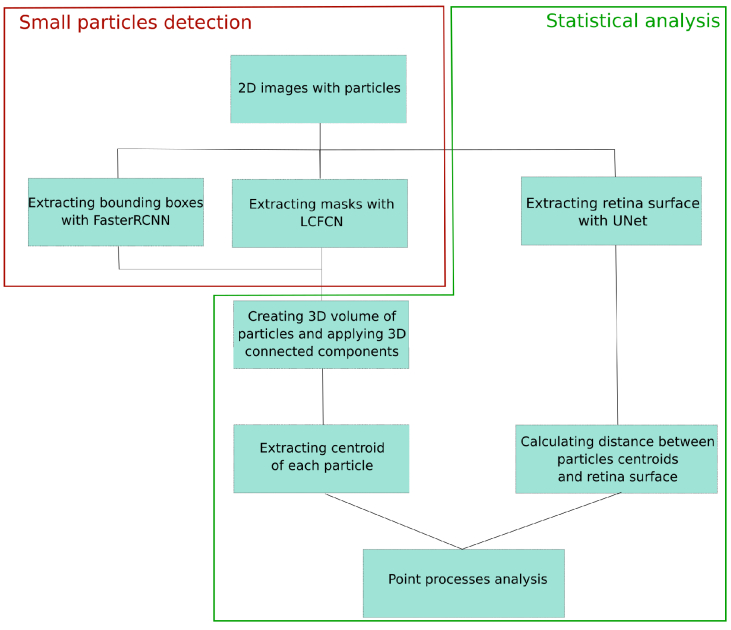
Flowchart illustrating the sequential stages and processing steps employed in the study.

The full pipeline used for small particles detection and statistical analysis of their distribution is summarized in Fig. 8.

## 3. Results

The present section details the results obtained for all three components of our proposed work, which were developed to demonstrate effective tools for the automated quantification of uveitis in OCT images of murine retina. By evaluating the performance of these tools across multiple metrics, we aim to demonstrate their effectiveness in accurately detecting and quantifying uveitis in OCT images.

### 3.1 OCT image classification

Prior approaches for the grading or classification of uveitis in OCT images relied on the assessment of the presence or absence of distinct particles [40]. Nevertheless, during the initial phases of disease progression, images of eyes that will subsequently exhibit uveitis show resemblance to those of healthy eyes, while in other instances, healthy retinas manifest particle presence at day 0 [41]. This renders differentiation based solely on such approach a challenging task.

We transferred 2 million disease-causing T cells into healthy mice of the C57BL/6 strain on day 0. This triggered the development of experimental autoimmune uveitis in the mice. To track the progression of the disease, we used OCT to obtain images of the mice’s eyes. We took images from two separate groups of mice at different time points: before the T cell transfer (day 0) and then on days 2, 6, and 14 after the transfer. We obtained from each retina a 3-D image with 512 2-D slices.

#### 3.1.1 Binary classification:

Our database comprises a set of 19 mouse retinas, acquired sequentially at day0, day2, day6, and day14, during the course of disease evolution. Specifically, day0 samples represent healthy retinas, while the subsequent scans correspond to different stages of the disease. Each retina consists of 512 2-D frames of size 1024×512 . Our primary objective is to perform binary classification of the disease by distinguishing days with uveitis (i.e., day2, day6, and day14) from the initial day. For this purpose, we utilize Efficient-Net7 as our underlying architecture, performing two-by-two classification, targeting day 0 - day 2, day 0 - day 6, and day 0 - day 14, respectively. We maintain the original images without any pre-processing, except for resizing each frame to 600×300 to match the model’s input shape. To improve generalization performance, we apply data augmentation techniques, such as random vertical flips, random zooms between 0 and 10%, and random rotations relative to the centre, within an angle range of 0 to $7^{\circ }$ .

To optimize the model’s performance, we train it with a batch size of 5, using the Adam optimizer with a learning rate of $10^{-4}$ [42], and binary-cross entropy as the loss function. Due to the limited amount of data available, we employ a cross-validation technique on retinas. Specifically, in each experiment, we select 17 retinas from each day for training and reserve 2 retinas for testing. This is motivated by the fact that images from the same retina may resemble each other. To prevent over-fitting, we perform out-of-sample testing at the retinas level.

In particular, we use the entire original images, we take two healthy retinas on day 0 and another two with uveitis for different days, one by one. For each case of classification, we run 5 separate experiments and we calculate the accuracy of classification (Eq. (7)). The metrics obtained are summarized in Table 1 for further analysis and interpretation.

(8) $$\text{Accuracy}=\frac{\text{True positives}+\text{True negatives}}{\text{Total number of images}}$$

(9) $$\text{Sensitivity}=\frac{\text{True positives}}{\text{True positives}+\text{False negatives}}$$

(10) $$\text{Specificity}=\frac{\text{True negatives}}{\text{True negatives}+\text{False positives}}$$

**Table 1.** Mean and standard deviation of accuracy, sensitivity and specificity obtained from 5 experiences for each case on original images.

Days	Day0-Day2	Day0-Day6	Day0-Day14
mean of accuracy ± std	0.8058 ± 0.071	0.94 ± 0.089	0.972 ± 0.01
mean of sensitivity ± std	0.794 ± 0.12	0.9374 ± 0.127	0.984 ± 0.023
mean of specificity ± std	0.834 ± 0.075	0.992 ± 0.014	0.964 ± 0.023

**Fig. 9.**
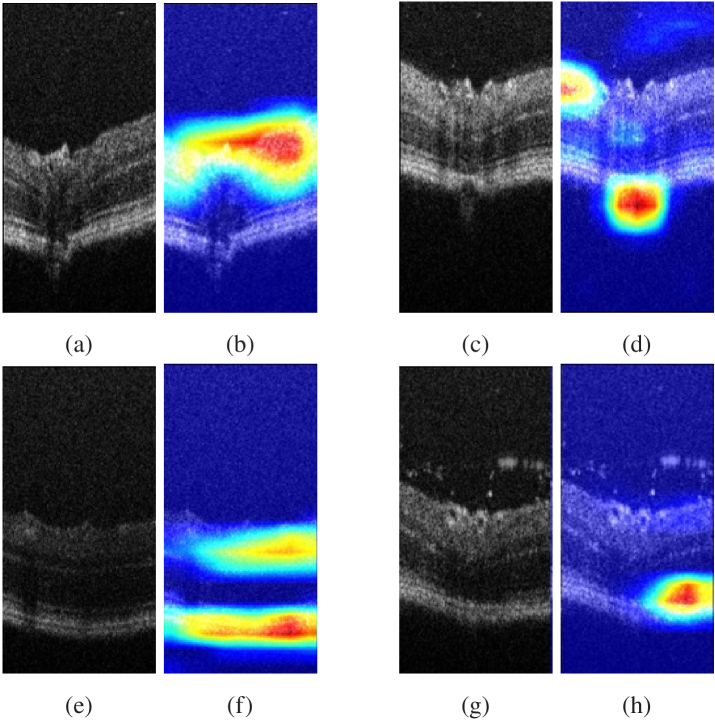
OCT images and their corresponding Grad-CAM outputs. (a) and (b) show the first image and its corresponding Grad-CAM output, while (c) and (d) show the second image and its Grad-CAM output.

**Fig. 10.**
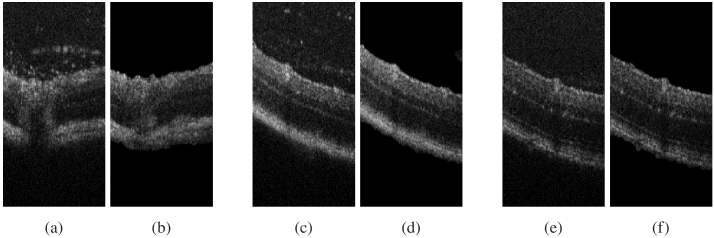
Original OCT images (a), (c), (e) and the extracted surface of the retina corresponding to each image (b), (d), (f).

After training the deep neural network, we utilized the Grad-CAM technique from Section 1 to provide interpretability and identify the important image regions that were most discriminative in the classification process. The Grad-CAM output for our images is illustrated in Fig. 9, where the Fig. 9(b), 9(d), 9(f) and 9(h) depicts the attention of the neural network. Notably, we observe that the network’s focus is consistently on the retina surface, even when the images contain particles, as evidenced by the image of Fig. 9(h). Hence, we proceeded to curate a new database by extracting the retina surface from the original dataset. An example of this process is demonstrated in Fig. 10. We replicated the training conditions outlined previously, with the exception of the database utilized. In this instance, we trained our network to perform classification solely on the extracted retinal images (Like images in Fig. 10(b), 10(d), 10(f)). The obtained results are presented in Table 2.

**Table 2.** Mean and standard deviation of accuracy, sensitivity and specificity obtained from 5 experiments for each case on images containing only retina surfaces.

Days	Day0-Day2	Day0-Day6	Day0-Day14
mean of accuracy ± std	0.84 ± 0.126	0.9099 ± 0.077	0.8895 ± 0.0288
mean of sensitivity ± std	0.826 ± 0.145	0.885 ± 0.125	0.972 ± 0.04
mean of specificity ± std	0.866 ± 0.139	0.9708 ± 0.06	0.8356 ± 0.062

#### 3.1.2 Multi-class classification:

**Fig. 11.**
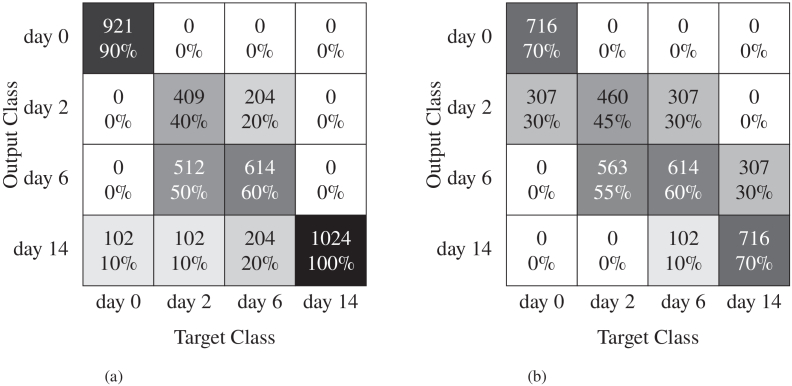
Confusion matrices for multi-day classification of original images. (b) Using a dataset of original images; (b) Using a dataset of images with retina surface only.

Previous work on grading systems for uveitis, based on 3D images, has relied on quantifying the number of particles [41]. In this study, we propose a CNN approach to classify disease progression across multiple time points simultaneously. We utilized the same network as in previous section (EfficientNet-B7), adapted the output layer to comprise four neurons, and implemented cross-entropy loss with soft-max as the output activation function. Our classes were based on the days of disease development, specifically day 0, day 2, day 6, and day 14. To ensure data balance, we utilized 17 retinas comprising 512 slices from each class, and employed identical pre-processing steps as in section 3.1.2. For testing, we set aside two retinas from each day. As for binary classification, we trained our model using two distinct databases, one containing original images and another containing only retina. The obtained accuracy for both scenarios is presented in Table 3. To further understand where the model struggled or became confused in the case of multi-class classification, confusion matrices for both cases are depicted in Fig. 11.

**Table 3.** Mean and standard deviation of accuracy obtained from 5 experiences on original images and images only with retina.

Database	Original images	Images with retina only
mean ± std	0.742 ± 0.0151	0.704 ± 0.0233

### 3.2 Small particles detection on 2-D OCT images:

In this study, we proposed three distinct methods for particle detection on 2-D images. Deep learning approaches differ in terms of their labeling and output capabilities. To begin with, we trained a Faster R-CNN model (Fig. 2), which is a supervised method that uses bounding boxes for detection. However, due to the substantial time required to annotate data for supervised learning, we suggest the use of a weakly supervised approach called LCFCN (Fig. 3), which only requires point annotations to obtain per-pixel segmentation of particles as output. In addition, we employed a MPP technique [32] that utilizes parameter fixation and retina surface masks to output bounding boxes around objects of interest.

The training of the first two methods was performed on a data-set of 250 2D OCT images, with bounding boxes and point annotations provided sequentially. We utilized stochastic gradient descent as an optimizer with a learning rate of $10^{-3}$ . For testing, we used 20 images that were not used during training and contained a considerable number of particles ( >10 ). The test images were labeled by two other specialists.

**Fig. 12.**
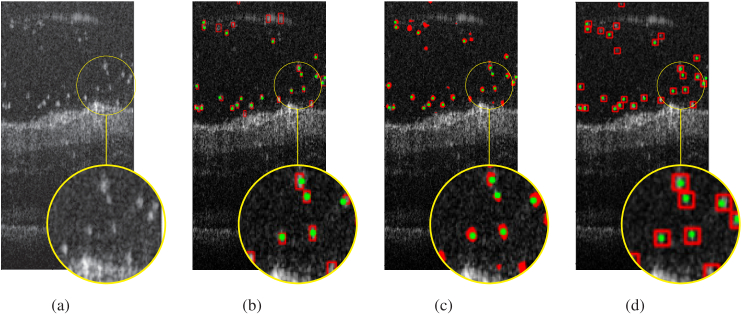
Different particle detection methods. (a) Original OCT images. (b) MPP method. (c) weakly supervised method. (d) supervised method. Green points on images represents annotation points or the ground truth. In red we have predictions (bounding boxes or masks).


To evaluate the performance of the particle detection methods using the same technique, we transformed the predicted bounding boxes into squared shape masks (segmentation), and used the center of ground-truth bounding boxes as the annotation point. We calculated the metrics using an existing software dAccuracy [43], which uses points as the ground truth and masks as the output. The metrics that we calculated included precision, recall, and the F1 Score, which is the harmonic mean of precision and recall. The obtained results are presented in Table 4 and Fig. 12).

(11) $$\begin{array}{r} \text{Precision}=\frac{TP}{TP+FP}, \end{array}$$

(12) $$\begin{array}{r} \text{Recall}=\frac{TP}{TP+FN}, \end{array}$$

(13) $$\begin{array}{r} \text{F1 score}=\frac{2\times TP}{2\times TP+FP+FN}\;, \end{array}$$

Where TP are true positives, FP are false positives and FN are false negatives.

**Table 4.** Results of particle detection using the MPP, supervised (F-RCNN), and weakly supervised (LCFCN) methods. Ground truth is given on the same dataset by two experts.

Method	Annotation of 1st expert			Annotation of 2nd expert		
	ObjectMPP	F-RCNN	LCFCN	ObjectMPP	F-RCNN	LCFCN
Precision	64.27%	**80.92** %	52.02%	61.55%	**68.49** %	57.79%
Recall	72.92%	86.13%	**94.71** %	89.21%	**95.94** %	81.43%
F1 score	67.98%	**82.75** %	66.73%	72.19%	**79.51** %	67.18%

#### 3.2.1 Particle counting:

**Fig. 13.**
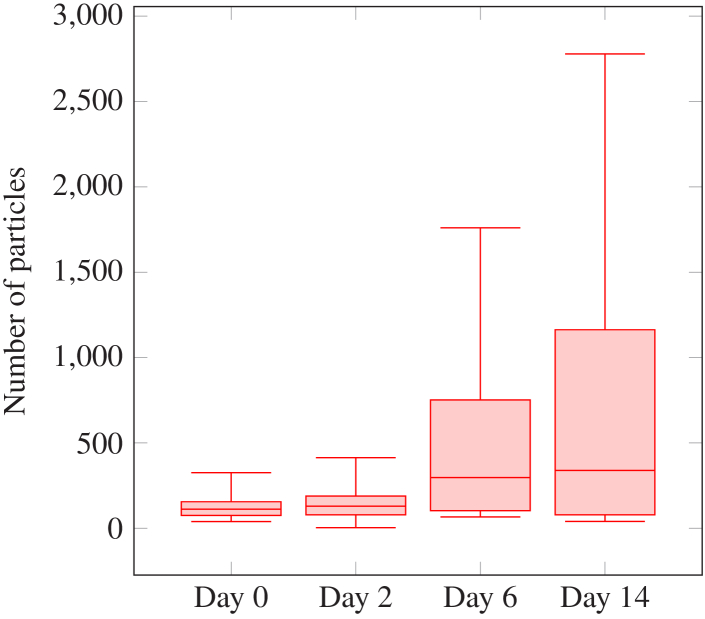
Box plot analysis of number of particles in retinas by days.

The LCFCN method was selected for particle detection due to its ability to generate per-pixel masks for accurate localization of particles, and subsequent refinement of metrics by discarding false positives via a bounding box provided by Faster R-CNN. To obtain a volumetric representation of detected particles, we reconstructed detections in 3-D by applying a connected components algorithm for labelling. In order to mitigate the effects of false positives, particles that were found to exist on only a single slice were eliminated from consideration. This step was based on the minimum expected size of a particle, as determined by the longitudinal resolution of the OCT system. We present the results of the number of particles between different days as a box plot in Fig. 13.

In the following subsection we investigate the distribution of uveitis particles and the variations in their numbers across different days, with respect to their distance from the retina.

#### 3.2.2 Measuring distance between particles and surface of the retina

**Fig. 14.**
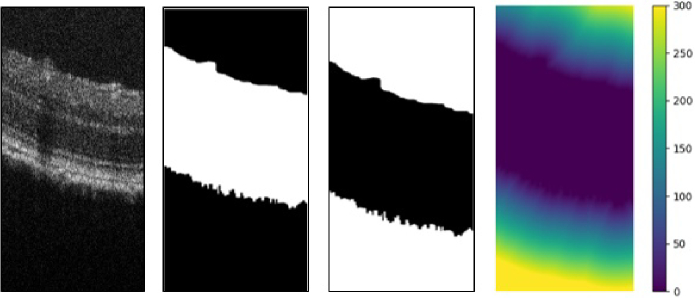
Measuring distance between particles and the surface of the retina. Images from left to right represent: the original image, extracted retina mask, negative values of the mask, and heat-map of the distance between each point and the retina surface.

To measure the distance between each particle and the surface of the retina, we adopted an alternative approach to the conventional perpendicular projection method. Specifically, we utilized the negative values of the retina mask to compute the distance between each point in the 3-D space and the nearest background point. In other words, the lowest distance between each point and the retina was calculated, thereby enabling accurate particle distance measurements. The distance value was subsequently extracted using the centroid coordinates of each particle. Figure 14 presents the results obtained after applying this method.

**Fig. 15.**
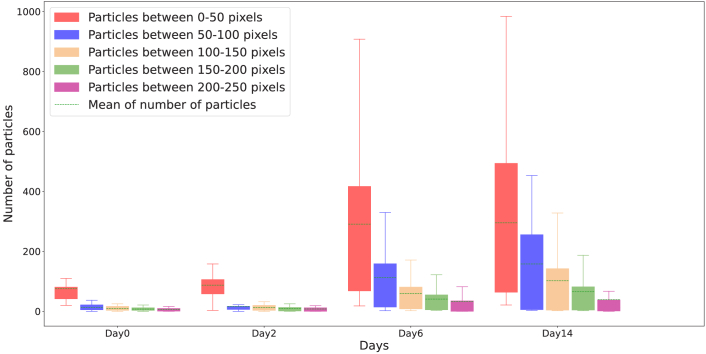
Box plot summarizing results comparing the distribution of different days in terms of the number of particles in each slice of distance from the retina surface.

Accurate measurement of the distance between the centroids of particles and the retina is a critical aspect in understanding the relationship between the particle distribution and the progression of the disease. To this end, Fig. 15 displays a box plot of the number of particles detected in each slice of the OCT image at various time points. By examining the distribution of particle counts over time, we can better comprehend how the disease manifests and progresses within the eye.

#### 3.2.3 Analysis of the distribution of particles in the vitreous above the retina:

**Fig. 16.**
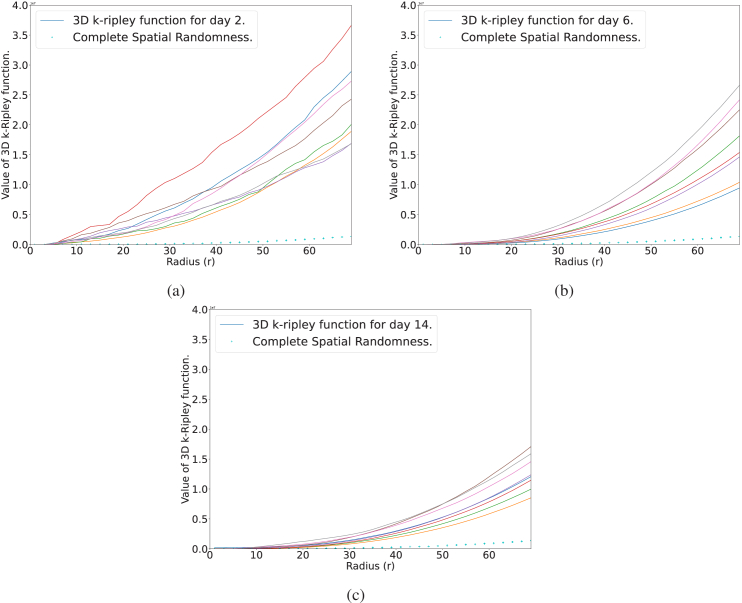
3D K-Ripley function for 8 different retinas of different days of evolution of the disease.

To analyse the spatial distribution of a group of points, we adopted the K-Ripley function [44], a popular method for spatial point pattern analysis. The K-Ripley function enables us to determine whether the observed point pattern is more or less clustered than expected from a given distribution. In our case, we compared the set of points with a random distribution. We applied the 3D K-Ripley function with edge correction, as described in [39], to the analysed point set. The study area was defined as a sphere with a radius equal to the width of the image, as shown in Fig. 7. The obtained results for different retinas on different days are presented in Fig. 16. The K-Ripley function plot for a given radius (r) between 0 and 70 pixels consistently exceeded the complete spatial randomness (CSR) plot, which is defined by $\frac{4\pi }{3}\times r^{3}$ , i.e. the entire volume of the sphere. This suggests that the uveitis particles exhibit a clustered pattern.

**Fig. 17.**
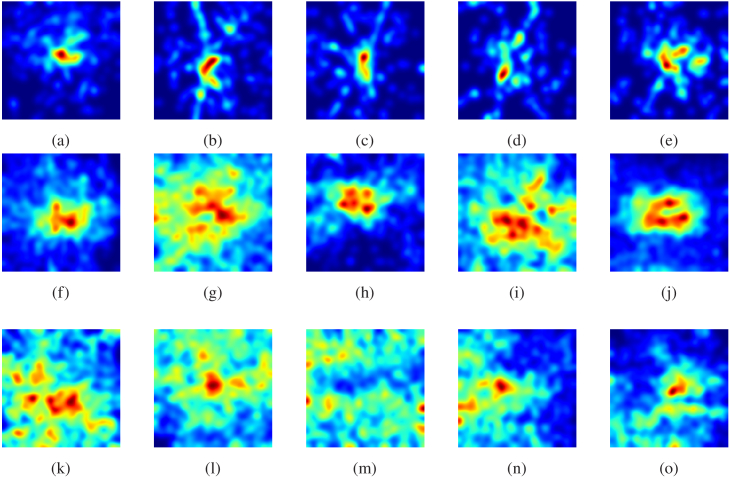
Heatmaps display the distribution of particles across different days, with the first row corresponding to day 2 (Images from (a) to (e)), the second row to day 6 (Images from (f) to (j)), and the third row to day 14 (Images from (k) to (o)).

The heatmaps of the particle distribution projected onto the retina surface (xy) axis are presented in Fig. 17. The results indicate that on day 2, the particles exhibited a higher degree of clustering in comparison to the other observed days. These findings are consistent with the higher values obtained for the K-function (Fig. 16) of particles on this particular day, thus providing evidence for the greater clustering tendency of particles during this period.

## 4. Discussion

### 4.1 Early detection of uveitis

The clinical assessment of uveitis is informed by OCT, but quantifying the extent of disease and applying this metric to therapeutic decision making remains a challenge [21] [45]. To address this challenge, we proposed the use of convolutional neural networks (CNNs) for the early detection of uveitis. Our model exhibits promising performance in differentiating between healthy and diseased retinal images. Specifically, in early stages of the disease, where clinical assessment of tissue state is difficult, our approach achieves up to 80% accuracy in binary classification between day 0 and other days, measured on a per-slice basis using 2D retinal images. To gain an overall understanding of the retina, we recommend utilizing a voting ensemble method. Our proposed CNN-based approach offers a novel solution to aid in the early detection and management of uveitis. Enabling interpretability in deep learning models is paramount to providing clinical intuition regarding the salient features of an OCT image. In this study, we employed the Grad-CAM technique, which generates visual explanations for model decisions. Our findings indicate that, at this time point, the model primarily discriminates between the two classes based on the analysis of the retinal surface and subsurface tissue. To validate this observation, we employed the same images utilized in previous experiments and isolated the segmentation of the retina, effectively removing all information above and below it. The model was then retrained on this reduced dataset. Encouragingly, our results demonstrate that the model can accurately classify retinal images using solely the retinal surface, with performance metrics comparable to the original experiments conducted on the full images. These findings highlight the potential utility of our approach for clinical decision-making and demonstrate the potential of interpretability techniques to enhance the understanding and usability of deep learning models in the medical field, in ophthalmology in particular. Expanding the scope of our classifier to encompass multiple classes allowed us to investigate the capability of the model to differentiate between different disease stages, as captured by 2D images of either the full retina or its surface. Our results indicate an accuracy ranging between 70% and 74% in this task. However, the model demonstrated some ambiguity in discerning between certain disease stages, which may be attributed to differences in the severity of the disease at different time points. Specifically, the response of the retina to the disease may vary in duration, leading to a delayed manifestation of its severity. To address this limitation, we suggest the development of a novel database that accounts for the gravity of the disease through the incorporation of histologic information. Such a database would enable the training of deep learning models with greater sensitivity and specificity, ultimately improving the diagnosis and management of uveitis.

### 4.2 Segmenting and counting particles in OCT

We used a weakly supervised method, LCFCN, which utilizes points as ground truth and outputs per-pixel segmentation of particles. We compared this approach to a well-established supervised object-detection method, Faster R-CNN, which employs bounding boxes as annotations. Additionally, we assessed a MPP technique that utilizes only the mask of the retina and a handcrafted parameter. Although the metrics of LCFCN were suboptimal due to false positives, we leveraged this approach for object detection by retaining only masks that were present in a bounding box generated by Faster R-CNN. This decision was motivated by the fact that LCFCN produces masks that are used to calculate the volume of each particle in 3D. We further annotated the 3-D reconstructed particles using the connected components algorithm, and removed particles present in only a single frame, based on the particle’s shape and the resolution of our OCT. We emphasize that our approach represents the first pipeline in the literature that utilizes deep learning to count the number of particles in a 3-D retina. Box plots of particle counts for each day suggest the possibility of finding particles in healthy retinas, while the number of particles shows significant variability during days 6 and 14. Notably, our findings indicate that the day cannot be accurately predicted based solely on the number of particles.

### 4.3 Segmenting retina surface and statistical analysis

To accurately classify retinas or study the distribution of particles with respect to the retina, a reliable segmentation of the retina surface is crucial. In this regard, we presented a novel method that employs fundamental image processing techniques to overcome the inherent noise present in mouse OCT retina images. While our approach has shown promise, we acknowledge its limitations, including the need to carefully select an appropriate threshold of binarization, given the considerable variability in grey levels observed between different retinas. To address this issue, we propose the use of a deep learning architecture, specifically a UNet, trained on well-generated masks derived from the image processing technique. Compared to our initial method, this new approach is visually more robust, produces better results, and eliminates the need to manually adjust parameters. To determine the spatial relationship between the centroids of the particles and the surface of the retina, we computed the shortest Euclidean distance between the non-zero points (i.e., those belonging to the particles) and the nearest zero point (i.e., background) on the retinal image. This method offers several practical advantages over direct projection onto the surface of the retina, as it avoids the potential for bias that may arise due to variations in the orientation of the retina in the image. In our final analysis, we investigated the spatial distribution of the centroids of particles as a point process, utilizing the 3D K-Ripley function with edge correction. The K-Ripley function was computed over a range of radii, and the resulting values were compared to those obtained for complete spatial randomness. Our analysis revealed a significant clustering effect of particles in 3D, as indicated by the deviation from the complete spatial randomness. It is providing important insights into the spatial organization of particles, which may have implications for understanding the underlying mechanisms of disease progression. However, it is important to note that this method is not capable of describing the movement or interaction of particles, and further studies should focus on these aspects to gain a more comprehensive understanding of the disease process.

## 5. Conclusion

We presented a fully automated framework for the evaluation and quantification of uveitis in OCT images of the mouse retina. Upon future adoption in humans, clinicians may be able to use it to speed up diagnostic as it enables the classification of 2D images into sick or healthy, even in the early stages of the disease. Moreover, we tried to justify neural network results by applying an explainable artificial intelligence method that provides a visual depiction of important features that our model uses to choose a class. The multi-class classification shows that deep learning can capture some characteristics that differentiate between days. We showed that important discriminative features peculiar to uveitis, are within the retina and not in the number of particles. Our framework can be used to detect, and extract centroids of particles in space to perform statistical analysis on the distribution of points, which in the future can prove beneficial to track particles and understand the evolution of the disease.

## Data Availability

The code for reproducing the results presented in this paper is available in [46], while the retinal OCT dataset used in this work can be downloaded from [47].
